# A Novel Label-Free Electrochemical Immunosensor for the Detection of Thyroid Transcription Factor 1 Using Ribbon-like Tungsten Disulfide-Reduced Graphene Oxide Nanohybrids and Gold Nanoparticles

**DOI:** 10.3390/molecules29020552

**Published:** 2024-01-22

**Authors:** Wenjing Wang, Huabiao Tang, Leiji Zhou, Zhaohui Li

**Affiliations:** 1 Zhengzhou Research Base, National Key Laboratory of Cotton Bio-Breeding and Integrated Utilization, School of Agricultural Sciences, Zhengzhou University, Zhengzhou 450001, China; wangwj@zzu.edu.cn; 2 College of Chemistry, Zhengzhou University, Zhengzhou 450001, China; tang@zzu.edu.cn; 3 Department of Chemistry, School of Chemistry and Chemical Engineering, Xiamen University, Xiamen 361005, China

**Keywords:** thyroid transcription factor 1, label-free, electrochemical immunosensor, tungsten disulfide, hydrogen peroxide

## Abstract

Thyroid transcription factor 1 (TTF1) is an important cancer-related biomarker for clinical diagnosis, especially for carcinomas of lung and thyroid origin. Herein, a novel label-free electrochemical immunosensor was prepared for TTF1 detection based on nanohybrids of ribbon-like tungsten disulfide-reduced graphene oxide (WS_2_-rGO) and gold nanoparticles (AuNPs). The proposed immunosensor employed H_2_O_2_ as the electrochemical probe because of the excellent peroxidase-like activity of ribbon-like WS2-rGO. The introduction of AuNPs not only enhanced the electrocatalytic activity of the immunosensor, but also provided immobilization sites for binding TTF1 antibodies. The electrochemical signals can be greatly amplified due to their excellent electrochemical performance, which realized the sensitive determination of TTF1 with a wide linear range of 0.025–50 ng mL^−1^ and a lower detection limit of 0.016 ng mL^−1^ (S/N = 3). Moreover, the immunosensor exhibited high selectivity, good reproducibility, and robust stability, as well as the ability to detect TTF1 in human serum with satisfactory results. These observed properties of the immunosensor enhance its potential practicability in clinical applications. This method can also be used for the detection of other tumor biomarkers by using the corresponding antigen–antibody complex.

## 1. Introduction

Thyroid transcription factor 1 (TTF1) is a tissue-specific transcription factor expressed mainly in the epithelial cells of the lungs and thyroid [1]. TTF1 immunopositivity has been used as a biomarker for the diagnosis of carcinomas of lung or thyroid origin by pathologists [2]. It has been reported that TTF1 is expressed in ∼90% of small cell lung cancers, ∼60–90% of lung adenocarcinomas and the majority of thyroid carcinomas, but is rarely expressed in other carcinomas [3]. Moreover, TTF1 is one of the best immunohistochemical biomarkers in the diagnosis of carcinomas of lung origin, especially lung adenocarcinoma [4]. Given its role in diagnosis, the detection of TTF1 is of vital importance. There have been many techniques developed for biomarker detection. However, the assays for TTF1 have rarely been reported, except for enzyme-linked immunoassay (ELISA) [5]. Although ELISA is common, it has some inherent disadvantages, such as its complex chromogenic procedure and strict time limitation [6]. Therefore, it is highly desirable to develop other simple and sensitive methods for TTF1 detection.

Over the past few years, electrochemical methods have been widely used in immunosensors due to their advantages, such as excellent specificity, good sensitivity and low cost [7,8,9]. Label-free electrochemical immunosensors in particular have attracted more attention, due to the ease involved with their operation, saving time and eliminating complex labeling processes when compared to sandwich-type immunosensors [10,11,12]. However, protein biomarkers cannot be used directly as an electrochemical indicator, because of the poor electrochemical properties of antigens and antibodies, as well as their complexity. Therefore, electrochemical probes were introduced to achieve label-free electrochemical immunosensing. Hydrogen peroxide (H_2_O_2_) is a common and important reactive oxygen species (ROS) involved in various biological and industrial processes. Monitoring its levels is essential for applications ranging from healthcare to environmental monitoring. In recent years, various electrochemical sensors have been developed to monitor H_2_O_2_ with high sensitivity and specificity [13,14]. Due to its excellent electrochemical response, H_2_O_2_ is widely employed as a probe in label-free electrochemical immunosensors [15,16].

In addition, to ensure sufficient sensitivity of electrochemical immunosensors, many efforts have been made to improve the electrochemical response towards the electrochemical probe by modifying various nanomaterials on the electrodes, such as carbon nanomaterials [17], metal and nonmetallic nano-oxides [18,19] and noble metal nanoparticles [20]. Amongst them, metal sulfides display great potential for signal amplification, due to their properties of good catalytic activity and good biocompatibility [21,22]. Tungsten disulfide (WS_2_) is a 2D-layered graphene-like material, which, due to its electrochemical properties, sheet-like architecture and high surface area, has attained much attention from researchers. Recently, WS_2_ has attracted significant attention due to its catalytic activity and superior thermal and oxidative stability, which make it a suitable candidate for electrochemical sensor applications [23,24]. However, the direct application of WS_2_ was limited due to its poor conductivity. To overcome this challenge, WS_2_ is usually incorporated with other materials such as graphene (Gr) which is known to exhibit excellent electrical conductivity and robust stability [25,26]. For instance, Yildirim et al. developed a molecularly imprinted electrochemical electrode based on WS_2_ nanoflowers on N,B-doped graphene, which demonstrated high selectivity and high sensitivity towards l-phenylalanine and made important contributions to the diagnosis of phenylketonuria [27]. Chen et al. reported a facile electrode based on mesoporous WS2 nanorods decorated with nitrogen-doped reduced graphene oxide networks. The modified electrode showed an excellent sensing ability towards a toxic antibiotic drug (roxarsone) [28]. In other studies, WS_2_-Gr nanohybrids were also utilized in Li-ion batteries [29], Na-ion batteries [30], UV photodetectors [31] and hydrogen evolution reactions [32]. To date, little has been reported about the application of WS_2_-Gr in electrochemical immunosensors.

**Scheme 1:**
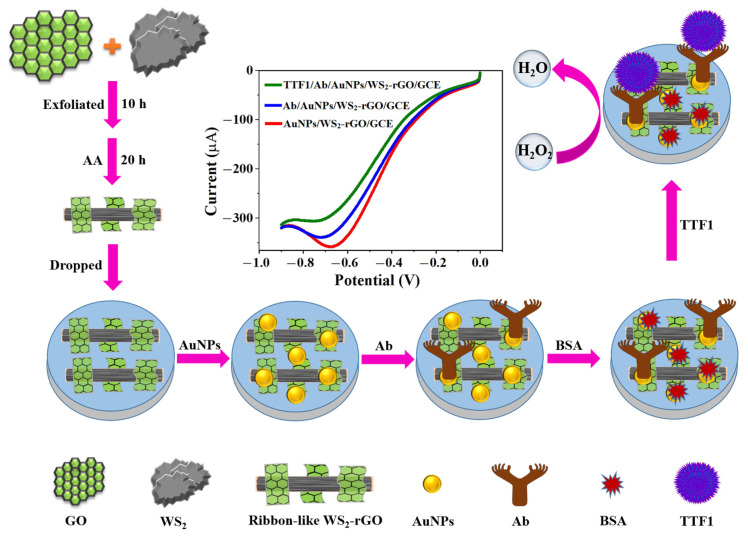
Schematic illustration of label-free electrochemical immunosensor for TTF1 detection.

In this work, a novel H_2_O_2_-based label-free electrochemical immunosensor for TTF1 determination was proposed utilizing ribbon-like WS_2_-rGO nanohybrids with intrinsic peroxidase-like activity (Scheme 1). The introduction of AuNPs in the fabrication of the immunosensor not only provided active sites to immobilize the antigens, but also further enhanced its electrochemical performance. The quantitative detection of TTF1 biomarkers could be achieved through the change in electrochemical current caused by the attachment of antigens and antibodies. Finally, the proposed electrochemical immunosensor was successfully used for the detection of TTF1 in a serum sample. This immunosensor has potential for the detection of other biomarkers and has great potential for practical applications.

## 2. Results and Discussion

### 2.1. Characterization of the WS_2_-rGO Nanohybrids

**Figure 1:**
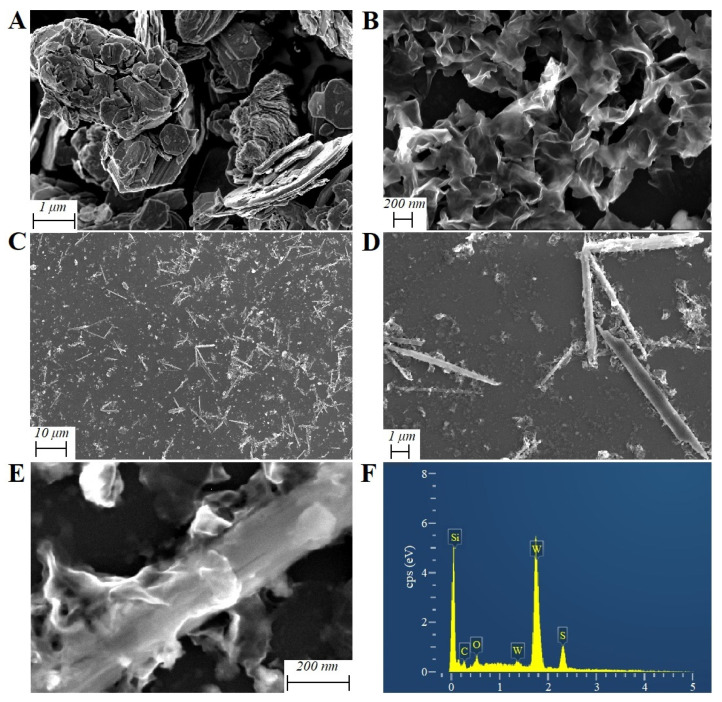
SEM images of bulk WS_2_ (**A**), rGO (**B**), WS_2_-rGO nanohybrids (**C**–**E**) and corresponding EDS analysis (**F**).

An SEM was used to observe the morphological features of the nanohybrids. Prior to mixing with GO, the natural WS_2_ showed an aggregation state with neat edges (Figure 1A). Moreover, the pure rGO, that was obtained through the reduction of GO without the presence of WS_2_, presented a film-like appearance with many random wrinkles and rugged edges (Figure 1B). When the mixture of WS_2_ and GO was exfoliated together using ultrasound and reduced by AA, WS_2_ presented a ribbon-like structure with a number of rGO nanofilms coated (Figure 1C). At a higher resolution, the structures of the ribbon-like WS_2_ and the adherent rGO nanofilms can be observed more clearly (Figure 1D,E). EDS analysis with C (9.23%), O (3.77%), W (13.58%) and S (22.52%) further confirmed the successful synthesis of WS_2_-rGO nanohybrids (Figure 1E). The Si element (50.91%) can also be observed, because the sample was placed on a silicon wafer for EDS analysis.

**Figure 2:**
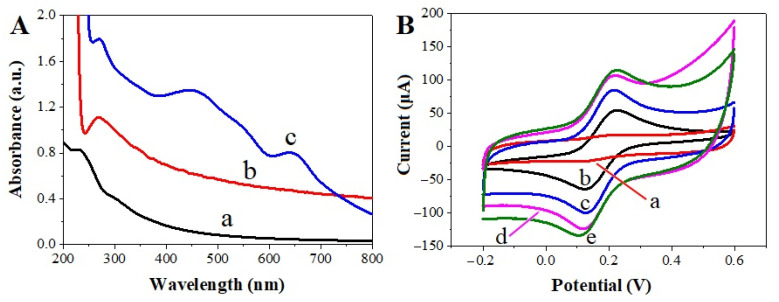
(**A**) UV-vis absorption spectra of GO (a), rGO (b) and WS_2_-rGO (c). (**B**) Cyclic voltammograms of K[Fe_3_(CN)_6_] (2.0 × 10^−3^ mol L^−1^) at the electrodes of GO/GCE (a), bare GCE (b), rGO/GCE (c), WS_2_-rGO/GCE (d) and AuNPs/WS_2_-rGO/GCE (e). Scan rate: 0.1 V s^−1^.

The obtained ribbon-like WS_2_-rGO nanohybrids were also analyzed using UV-vis spectra (Figure 2A). In the spectra of GO, a typical UV-vis absorption peak at about 230 nm and a shoulder peak at about 300 nm were observed, which corresponded to the π-π* transitions of the aromatic C=C band and the n-π* transitions of the C=O band, respectively (Figure 2A, curve a). After reacting with AA, the two peaks disappeared and a new absorption peak appeared at 270 nm (Figure 2A, curve b), which illustrated that the GO was reduced to rGO with AA. In the spectra of WS_2_-rGO nanohybrids, in addition to the absorption of rGO, new absorption peaks were observed in the region from 400 nm to 700 nm (Figure 2A, curve c). It has been reported that bulk WS_2_ has an absorption edge of 920 nm [33]. However, the UV-vis absorption exhibits a significant blue shift when the size decreases from bulk WS_2_ to nanoscale [34]. The UV-vis absorption in this work further proved the successful synthesis of WS_2_-rGO nanohybrids.

FTIR analysis was used to confirm the effective non-covalent modification of functional groups and estimate the vibrational mutations of the molecules. Figure S1 illustrates the FTIR spectrum of WS_2_ and the WS_2_-rGO nanocomposite. For WS_2_, the vibrational band at 3516 cm^−1^ can be assigned to atmospheric –OH, and the 2921 cm^−1^ band can be associated with W-S bending vibrations. The bands found at 1639 cm^−1^ and 1576 cm^−1^ can be ascribed to the stretching vibrations of the hydroxyl groups. The bands positioned at 1096 cm^−1^ and 583 cm^−1^ are attributed to S-S and W-S bonds, respectively. For WS_2_-rGO, the bands appearing at 615 cm^−1^ and 490 cm^−1^ can be ascribed to C-S and S-S stretching vibrations. Most of the characteristic bands associated with WS_2_ are observed in the WS_2_-rGO composite, with a partial shift in the band positions. The 2921 cm^−1^ and 2919 cm^−1^ bands assigned to W-S in WS_2_ and the WS_2_-rGO composite, and the 615 cm^−1^ band attributed to C-S in WS_2_-rGO detected thus confirm the formation of the composite. Significantly, the FTIR spectrum results established the composition and interaction of ribbon-like WS_2_ on the surface of the rGO sheets.

To evaluate the electrochemical performance of the nanohybrids, the responses of various electrodes were analyzed using cyclic voltammetry (CV) in 1 × 10^−3^ mol L^−1^ [Fe(CN)_6_]^3−/4−^ containing a 0.1 mol L^−1^ KCl solution. Before modification, a pair of typical reversible redox peaks was observed on bare GCE (Figure 2B, curve b). The redox peaks almost disappeared on GO/GCE, due to the poor conductivity of GO (Figure 2B, curve a). After being modified with rGO, the redox peaks on rGO/GCE increased sharply (Figure 2B, curve c) due to the good conductivity of rGO, which further illustrated that GO was successfully reduced with AA. When GCE was modified with the WS_2_-rGO nanohybrids, the redox peaks and charging current all increased further (Figure 2B, curve d), benefitting from the excellent electrical conductivity and the large specific area of WS_2_-rGO. Finally, the redox currents of [Fe(CN)_6_]^3−/4−^ reached their maximum on AuNPs/WS_2_-rGO/GCE because of the introduction of AuNPs (Figure 2B, curve e), which confirmed that the electrode was assembled successfully and could be used for further analysis.

### 2.2. Electrochemical Behavior of the Immunosensor

**Figure 3:**
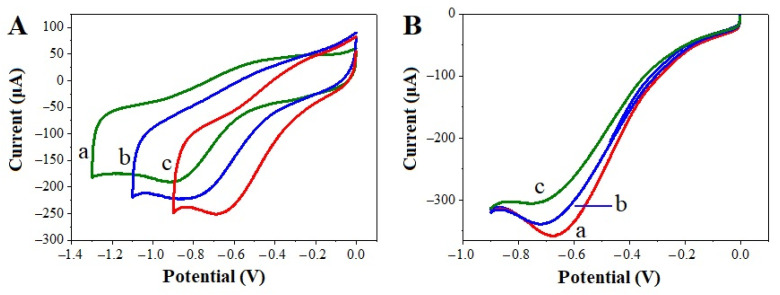
(**A**) CVs of rGO/GCE (a), WS_2_-rGO/GCE (b) and AuNPs/WS_2_-rGO/GCE (c) in the presence of 5 mM of H_2_O_2_ in N_2_-saturated pH 7.0 PBS. Scan rate: 0.1 V s^−1^. (**B**) LSVs of AuNPs/WS_2_-rGO/GCE (a), Ab/AuNPs/WS_2_-rGO/GCE (b), and TTF1/Ab/AuNPs/WS_2_-rGO/GCE (c) in the presence of 5 mM of H_2_O_2_ in N_2_-saturated pH 7.0 PBS. Scan rate: 0.1 V s^−1^.

In general, the sensitivity of the label-free electrochemical immunosensor depends on the electrochemical activity of the biosensor towards the electrochemical probe. We therefore investigated the electrocatalytic activity of the ribbon-like WS_2_-rGO and AuNPs nanohybrids towards H_2_O_2_. In the absence of H_2_O_2_, there were no peaks on all of the modified electrodes (Figure S2A–F). When 5 mM of H_2_O_2_ was added into the N_2_-saturated PBS, there was still no obvious response on bare GCE (Figure S2G, curve a), whereas a slight reduction in peak was observed on GO/GCE (Figure S2G, curve b). After coating GCE with rGO, the electrochemical response towards H_2_O_2_ was greatly increased, due to the good conductivity of rGO (Figure S2G, curve c). Additionally, when WS_2_ was doped on GCE, the electrochemical response towards H_2_O_2_ increased more sharply than that of bare GCE (Figure S2H). Moreover, with the introduction of nano-WS_2_, the reduction current was further increased compared to that of rGO/GCE (Figure 3A, curves a and b), indicating that the WS_2_-rGO nanohybrids had good electrocatalytic activity towards H_2_O_2_. The electrochemical properties reached optimum results on AuNPs/WS_2_-rGO/GCE (Figure 3A, curve c), which was a result of the comprehensive effect of nano-WS_2_, rGO and AuNPs. The above result further confirmed that the catalytic activity of AuNPs/WS_2_-rGO nanocomposites was exceptional in the electrochemical reduction of H_2_O_2_, which made it possible to construct a highly sensitive immunosensor.

The fabrication process of the label-free electrochemical immunosensor was investigated using linear sweep voltammetry (LSV). Before the immobilization of anti-TTF1, a well-defined reduced peak was observed in AuNPs/WS_2_-rGO/GCE (Figure 3B, curve a). After the modified electrode was immobilized with anti-TTF1 and blocked using BSA, the electrochemical response towards H_2_O_2_ decreased gradually (Figure 3B, curve b), which suggested that the non-conductive substances of anti-TTF1 and BSA can hinder electron transfer and disturb the electrocatalytic activity toward H_2_O_2_. The peak current of H_2_O_2_ was further decreased when TTF1 was captured with Ab/AuNPs/WS_2_-rGO/GCE (Figure 3B, curve c), which indicated that the complex of antibodies and antigens can further disturb the reduction of H_2_O_2_ on the surface of the immunosensor. The gradual decrease in the response current indicated that the label-free electrochemical immunosensor was prepared successfully and can be used for TTF1 detection with H_2_O_2_ acting as the electrochemical probe.

### 2.3. Optimization of Detection Conditions

In order to achieve an optimal electrochemical signal response, the optimization of experimental conditions is necessary. The pH value can influence the electrocatalytic process of AuNPs/WS_2_-rGO/GCE. Figure S3 shows the electrocatalytic current responses of AuNPs/WS_2_-rGO/GCE in PBS at different pH values in 5 mM of H_2_O_2_. As shown in this figure, the optimal electrochemical signal response was achieved at a pH of 7.0. Moreover, the antibodies and antigens can maintain their biological activity in this approximately neutral condition. Therefore, PBS at pH 7.0 was selected for use throughout this study.

**Figure 4:**
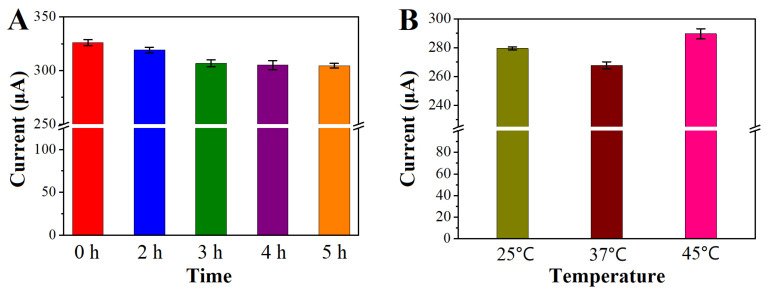
Optimization of (**A**) incubation time of anti-TTF1 on AuNPs/WS_2_-rGO/GCE and (**B**) incubation temperature of TTF1 antigens on BSA/Ab/AuNPs/WS_2_-rGO/GCE. LSV responses were performed in a N_2_-saturated pH 7.0 PBS containing 5 mM of H_2_O_2_. Error bars represent the standard deviation (SD) from three independent determinations.

In order to achieve the optimal electrochemical result, parameters such as the incubation time of anti-TTF1 and the incubation temperature of TTF1 antigens were also studied. As shown in Figure 4A, when the incubation time of anti-TTF1 was varied from 0 to 4 h, the response currents of H_2_O_2_ decreased gradually, as a result of more anti-TTF1 molecules being conjugated to the surface of AuNPs/WS_2_-rGO/GCE. The reduction current of H_2_O_2_ was observed to be constant as the incubation time was increased from 4 h to 8 h, indicating that the anti-TTF1 had reached saturation on the electrode. Therefore, 4 h was used as the optimum incubation time for anti-TTF1 conjugation. For more efficient binding of TTF1 antigens with anti-TTF1, the incubation temperature of TTF1 on the surface of Ab/AuNPs/WS_2_-rGO/GCE was also investigated. As shown in Figure 4B, the greatest reduction in electrochemical response was observed at 37 °C, demonstrating that 37 °C is more suitable for the combination of TTF1 antigens with anti-TTF1.

### 2.4. Electrochemical Detection of TTF1 Antigens

**Figure 5:**
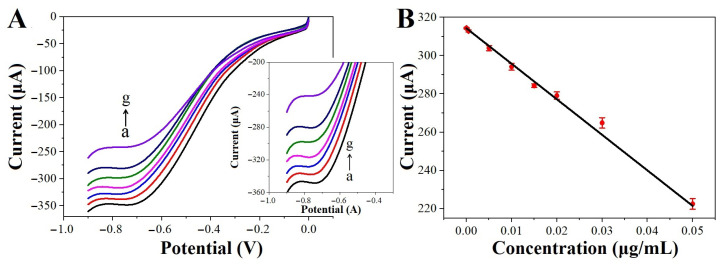
(**A**) LSV responses of the immunosensor in N_2_-saturated pH 7.0 PBS containing 5 mM of H_2_O_2_ for TTF1 detection with different concentrations. From a to g: 0.025 ng mL^−1^, 0.05 ng mL^−1^, 5 ng mL^−1^, 10 ng mL^−1^, 20 ng mL^−1^, 30 ng mL^−1^ and 50 ng mL^−1^. Scan rate: 0.1 V s^−1^. (**B**) The corresponding calibration curve. Error bars represent the SD from three independent determinations.

Under optimal conditions, the electrochemical immunosensing system developed was used for the detection of TTF1 antigens using LSV. As the concentration of TTF1 was increased from 25 pg mL^−1^ to 50 ng mL^−1^, the amount of TTF1 antigens conjugated to the surface of Ab/AuNPs/WS_2_-rGO/GCE also increased, leading to the gradual decrease in electrochemical responses (Figure 5A). Moreover, a good linear relationship was obtained between the concentration of TTF1 and the reduction current of H_2_O_2_ (*i*_P_ (μA) = −1857.63 c (μg mL^−1^) + 314.32 (R = 0.9940)) with a detection limit of 0.016 ng mL^−1^ (Figure 5B). Compared with the reported ELISA methods [5], a wider linear range was obtained in this work. The good sensitivity observed benefitted from the signal amplification strategy of the AuNPs/WS_2_-rGO nanocomposites. The results indicated that the electrochemical immunosensor developed could be employed for the ultra-sensitive detection of TTF1 antigens.

### 2.5. Specificity, Reproducibility and Stability of the Immunosensor

Specificity is one of the most important indexes of the label-free electrochemical immunosensor. The selectivity analysis was performed using thrombospondin-1 (TSP1) [35], extracellular signal-regulated kinase (ERK) [36], BSA and glucose (GLU) as interference. A 15 ng mL^−1^ of TTF1 solution, or a mixture of 15 ng mL^−1^ of TTF1 with 100 ng mL^−1^ of interference solution, was detected using the developed immunosensor of Ab/AuNPs/WS_2_-rGO/GCE. As shown in Figure S4A, the electrochemical response of H_2_O_2_ on the immunosensors that were incubated in the mixtures was almost the same as that on TTF1/Ab/AuNPs/WS_2_-rGO/GCE, suggesting that the interference of TSP1, ERK, BSA and GLU did not disturb the detection of TTF1. This result proved the high specificity of the developed immunosensor.

To test the reproducibility of this assay, the H_2_O_2_ response was studied on five independent immunosensors. The reduction currents of H_2_O_2_ were almost the same, with a relative standard deviation (RSD) of 1.02% (Figure S4B). To assess the assay’s repeatability, identical samples of H_2_O_2_ were tested using the same immunosensor. The reduction currents of H_2_O_2_ exhibited remarkable consistency, with a low RSD of 1.06%, affirming the high reproducibility of this measurement. Additionally, the response current of the electrochemical immunosensors after storage at 4 °C for 7 days was observed to have only decreased by 4.30%, as compared to that of the freshly prepared immunosensor. The above results indicated good reproducibility and stability of the developed immunosensor.

### 2.6. Determination of TTF1 in Human Serum Sample

In order to evaluate the viability of the developed method, the label-free electrochemical immunosensor of Ab/AuNPs/WS_2_-rGO/GCE was used for the detection of TTF1 in a human serum sample. The measurement method of TTF1 in serum was the same as that used in the standard TTF1 solution. Subsequently, a certain concentration of TTF1 was added into the serum sample, and the final content of TTF1 in the samples was measured afterwards. As shown in Table S1, the concentration of TTF1 in the serum sample was obtained with a concentration of 0.76 ng mL^−1^, which was found to be within the range of reported values [37]. Moreover, the results were observed to range from 98.7% to 103.1%, with an RSD lower than 5.2%, suggesting the acceptable accuracy of the developed immunosensor. Finally, we compared the proposed electrochemical immunosensor with the ELISA method to measure TTF1 content in serum samples. The results showed that the TTF1 levels measured with these two methods are essentially consistent (Table S1), indicating the reliability of the method proposed in this work.

## 3. Materials and Methods

### 3.1. Apparatus and Reagents

A CHI650B electrochemical platform (Chenhua Instrument Co., Ltd., Shanghai, China) was employed to observe the electrochemical behaviors. A traditional three-electrode system was used for the electrochemical measurements, with a bare glassy carbon electrode (GCE, 4 mm in diameter) or a modified GCE as the working electrode, a platinum wire as the auxiliary electrode and a saturated calomel electrode (SCE) as the reference electrode. A Scientz-IID ultrasonic cell crusher (Xinzhi biotechnology co., Ltd., Ningbo, China) was used to exfoliate the GO or the mixture of GO and WS_2_. The characterization of the materials was performed using a scanning electron microscope (SEM, Zeiss, sigma 500, Jena, Germany), UV-2550 spectrophotometer (Shimadzu, Kyoto, Japan) and Fourier-transform Infra-Red spectra (FTIR, PerkinElmer, Waltham, MA, USA).

TTF1 antibodies (anti-TTF1 or Ab) and TTF1 antigens were purchased from Hunan Jingke biotechnology Co., Ltd., Changsha, China. The commercial ELISA kits were purchased from Cusabio (Wuhan, China). Bulk WS_2_, graphite powers and HAuCl_4_·3H_2_O were purchased from Shanghai Macklin Biochemical Co., Ltd., China. Ascorbic acid (AA) was purchased from Shanghai Aladdin Chemistry Co., Ltd., China. NaOH, N,N-Dimethylformamide (DMF) and hydrogen peroxide (H_2_O_2_, 30%) were purchased from Tianjin Fuchen chemical reagent Co., Ltd., China. The nitrogen-saturated phosphate buffer solution (N_2_-saturated PBS, pH 7.0) was prepared by mixing a 0.1 mol L^−1^ NaH_2_PO_4_ and Na_2_HPO_4_ solution and removing the dissolved oxygen by purging nitrogen gas for 20 min. The water used was ultra-pure deionized water (18.2 MΩ·cm).

The venous serum samples were obtained from a clinical laboratory and approved by the Ethics Committee of Zhengzhou Hospital of Traditional Chinese Medicine (Ethics ID: AF/SC-08/03.0). All sample donors provided voluntary and informed consent.

### 3.2. Synthesis of Ribbon-like WS_2_-rGO Nanohybrids

Graphene oxide (GO) was synthesized from graphite powder using Hummers’ method, as described in the previous report [38]. In order to synthesize WS_2_-rGO nanohybrids, 10 mg of bulk WS_2_ and 10 mg of GO powder were dispersed in 10 mL of DMF. The mixture was then exfoliated using an ultrasonic cell crusher with a power of 855 W. After ultra-sonicating for 10 h, the homogeneous solution was set for 24 h at room temperature to remove the WS_2_ and GO crowds that were not exfoliated. Then, the supernatant was centrifuged at 1000 g to further remove the bulk WS_2_ and GO. The reduction of GO was performed by adding 100 mg of reductant AA to the supernatant mixture of GO and WS_2_ that was obtained through centrifugation [39]. After heating for 20 h at 95 °C with vigorous stirring, the rGO was generated with doped nano-WS_2_. Then, the obtained WS_2_-rGO nanohybrids were washed several times using alcohol and water, until the pH reached 7.0. Finally, the WS_2_-rGO nanohybrids were freeze-dried and stored at 4 °C for further use. For comparison, the pure rGO was also synthesized this way, but without the presence of WS_2_.

### 3.3. Preparation of Electrochemical Immunosensor

Firstly, 1 mg of WS_2_-rGO nanohybrids was dispersed in 1 mL of DMF and ultra-sonicated for 30 min. Before fabrication, the bare GCE was polished to a mirror-like finish using 0.05 μm of alumina powder. As shown in Scheme 1, the electrochemical immunosensor was fabricated by doping 10 μL of WS_2_-rGO suspension onto the GCE surface and then air drying to prepare a WS_2_-rGO-modified GCE (WS_2_-rGO/GCE). In order to conjugate anti-TTF1, AuNPs were coated on the surface of WS_2_-rGO/GCE using electrodeposition for 30 s at a potential of −0.2 V in a 1% HAuCl_4_ solution (noted as AuNPs/WS_2_-rGO/GCE). After washing with PBS (pH = 7.0), 6 μL of 10 ng mL^−1^ anti-TTF1 was dropped on the AuNPs/WS_2_-rGO/GCE surface and incubated at 4 °C for 4 h. This was then followed by incubation with 6 μL of 1% BSA for 1 h to eliminate the non-specific adsorption (noted as Ab/AuNPs/WS_2_-rGO/GCE). After each incubation, the immunosensor was washed with PBS (pH = 7.0) to remove the unbound substances. Thereafter, the prepared immunosensor Ab/AuNPs/WS_2_-rGO/GCE was ready for the detection of TTF1 antigens.

### 3.4. Electrochemical Measurements of TTF1

In order to achieve the optimal electrochemical result, parameters such as the incubation time of anti-TTF1, the incubation temperature of TTF1 antigens and the optimal reaction pH were studied. To bond the TTF1 antigens, the prepared Ab/AuNPs/WS_2_-rGO/GCE was incubated with 6 μL of TTF1 antigens (with varying concentration in pH 7.0 PBS) for 1 h at 37 °C. Subsequently, the proposed electrochemical immunosensor (noted as TTF1/Ab/ AuNPs/WS_2_-rGO/GCE) was washed extensively to remove unbounded TTF1 molecules. Electrochemical measurement of the TTF1 biomarker was performed in a N_2_-saturated PBS (pH = 7.0) solution by adding 5 mM of H_2_O_2_ as a probe. After covering with TTF1 antigens, the resistance would increase and the response current of H_2_O_2_ would be decreased, which can be used as the signal to quantify TTF1 antigens.

## 4. Conclusions

In this work, we have developed a novel label-free electrochemical immunosensor for TTF1 detection using AuNPs/WS_2_-rGO nanocomposites as the electrode materials and H_2_O_2_ as the electrochemical probe, respectively. The ribbon-like WS_2_-rGO nanohybrids showed excellent electrocatalytic activity towards the electrochemical reduction of H_2_O_2_. The introduction of AuNPs not only enhanced the electrochemical performance of the immunosensor, but also provided active sites for anti-TTF1 immobilization. Through using UV-vis, FTIR and SEM characterizations, the structure and morphology of the ribbon-like WS_2_-rGO composite were analyzed. Taking advantage of the synergistic effect of nano-WS_2_, rGO and AuNPs, the label-free electrochemical immunosensor developed high sensitivity for TTF1 detection, with a wide linear range of 25 pg mL^−1^ to 50 ng mL^−1^ and a lower detection limit of 16 pg mL^−1^. Moreover, the immunosensor exhibited high selectivity, good reproducibility and robust stability, which enable it to be applied in human serum with satisfactory results. This method provided a potential application in clinical diagnosis and can be used for the detection of other biomarkers by using the corresponding antigen-antibody complex.

## Data Availability

Data are contained within the article and Supplementary Materials.

## Supplementary Materials

The following supporting information can be downloaded at: https://www.mdpi.com/article/10.3390/molecules29020552/s1.
