# Supplementary material for: A Novel Label-Free Electrochemical Immunosensor for the Detection of Thyroid Transcription Factor 1 Using Ribbon-like Tungsten Disulfide-Reduced Graphene Oxide Nanohybrids and Gold Nanoparticles
Source: Molecules. 2024 Jan 22;29(2):552. doi: 10.3390/molecules29020552 (PMC10819751; doi:10.3390/molecules29020552)
Supplement: Supplementary file 1 [file molecules-29-00552-s001.zip › molecules-2737661-supplementary.pdf]

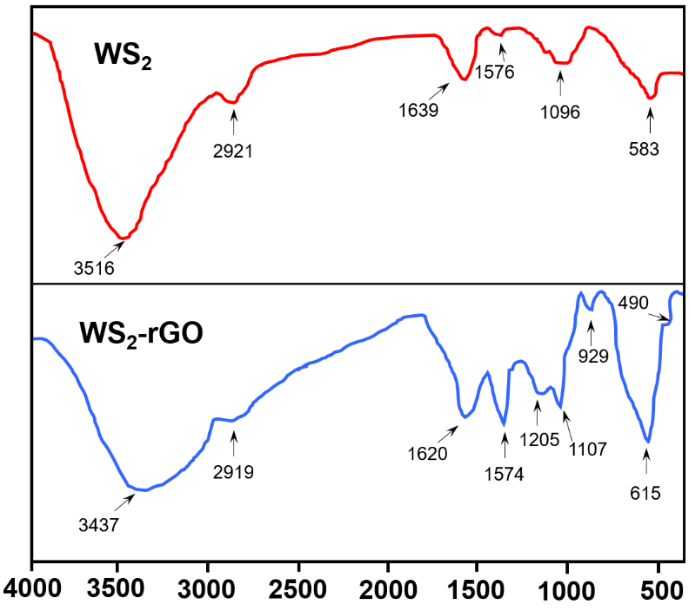


**Figure S1.** FTIR analysis of $WS_2$ and $WS_2$-rGO.

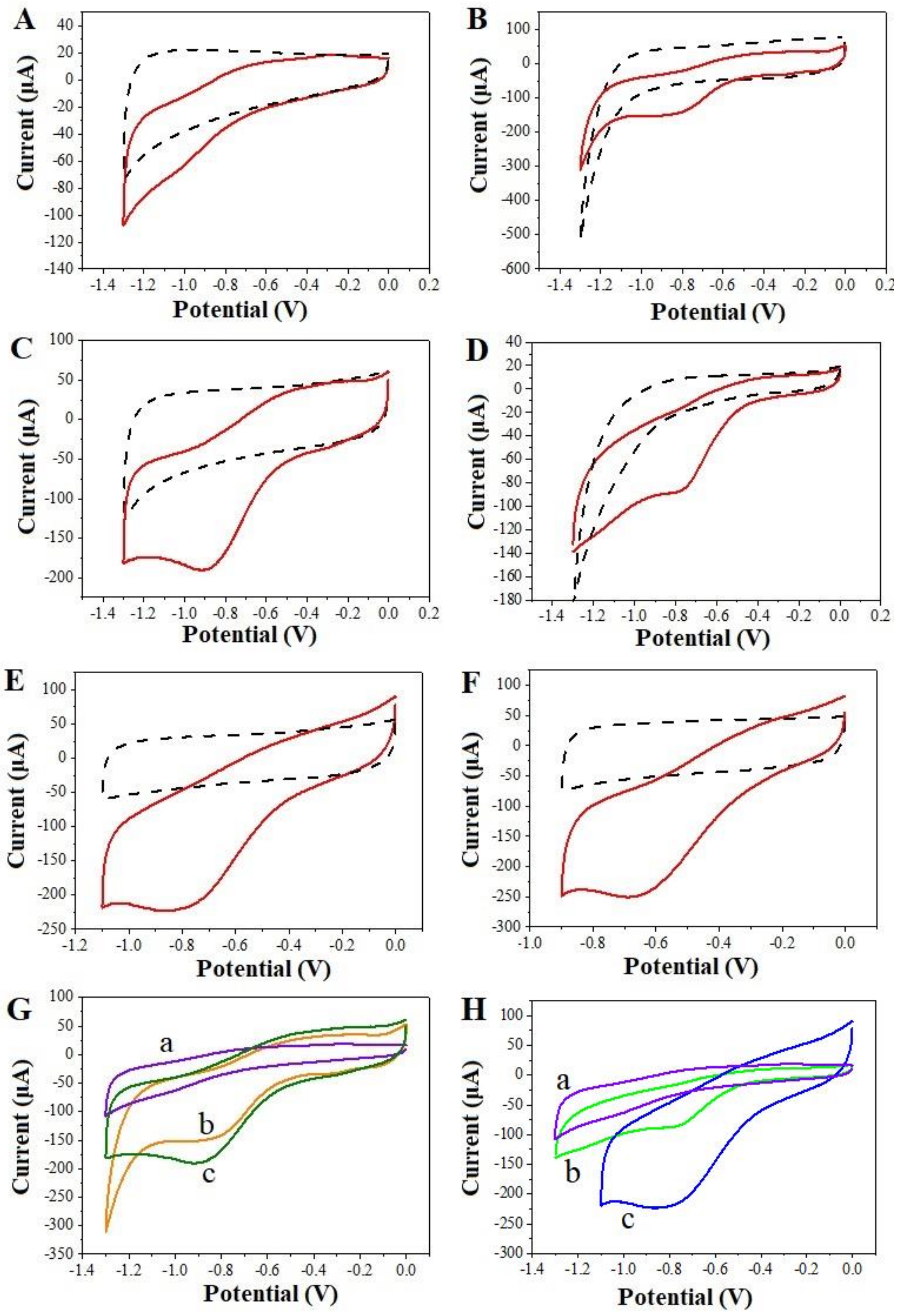


**Figure S2.** CVs of bare GCE (**A**), GO/GCE (**B**), rGO/GCE (**C**), $WS_2$/GCE (**D**), $WS_2$-rGO/GCE (**E**) and AuNPs/$WS_2$-rGO/GCE (**F**) in the absence (dotted lines) and in the presence (solid lines) of 5 mM $H_2O_2$ in $N_2$-saturated PBS. (**G**) CVs of bare GCE (a), GO/GCE (b) and rGO/GCE (c) in the presence of 5 mM $H_2O_2$ in $N_2$-saturated PBS. (**H**) CVs of bare GCE (a), $WS_2$/GCE (b) and $WS_2$-rGO/GCE (c) in the presence of 5 mM $H_2O_2$ in $N_2$-saturated PBS.

Scan rate: 0.1 V $s^{-1}$

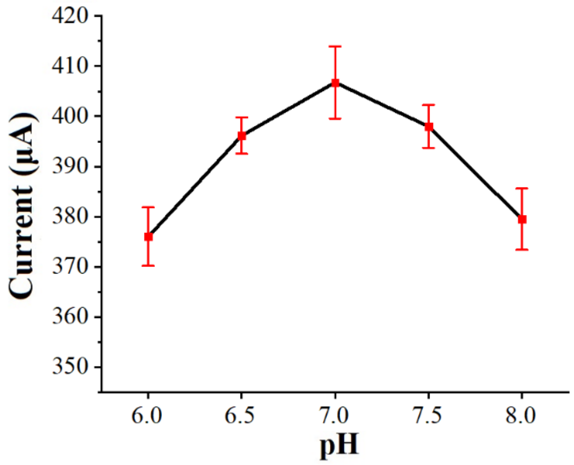


**Figure S3.** Electrocatalytic current responses of AuNPs/$WS_2$-rGO/GCE in PBS at different pH values towards the reduction of 5 mM $H_2O_2$.

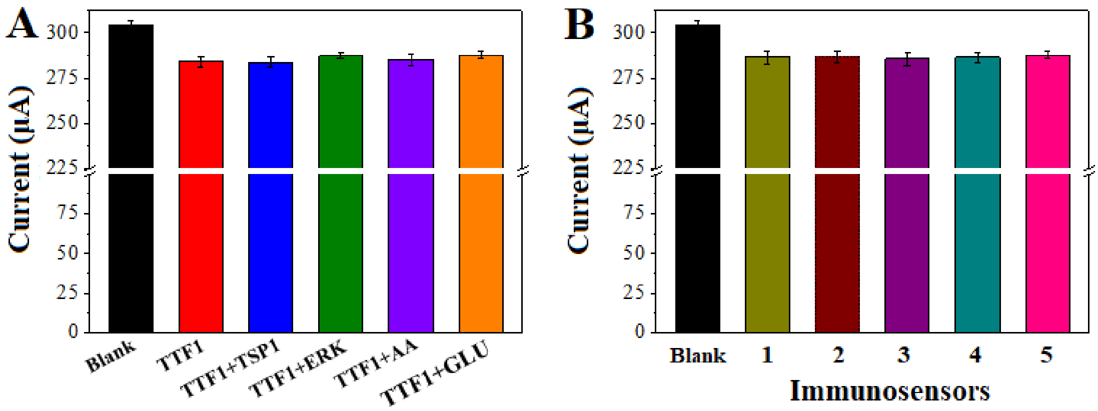


**Figure S4.** (**A**) Electrochemical signal responses of the immunosensor towards blank PBS, 15 ng $mL^{-1}$ TTF1, 15 ng $mL^{-1}$ TTF1 +100 ng $mL^{-1}$ TSP1, 15 ng $mL^{-1}$ TTF1 +100 ng $mL^{-1}$ ERK, 15 ng $mL^{-1}$ TTF1 + 100 ng $mL^{-1}$ BSA and 15 ng $mL^{-1}$ TTF1 +100 ng $mL^{-1}$ GLU.

(**B**) Electrochemical signal responses of the five immunosensors independently fabricated towards 15 ng $mL^{-1}$ TTF1. LSV responses were performed in a $N_2$-saturated pH 7.0 PBS containing 5 mM $H_2O_2$. Error bars represent SD from three independent determinations.

**Table S1.** Determination of TTF1 in human serum sample.

| | Initial TTF1 in Serum Sample (ng mL$^{-1}$) | Added TTF1 (ng mL$^{-1}$) | Measured TTF1 (ng/mL) | Recovery (%, n = 3) | RSD (%, n=3) |
|---|---|---|---|---|---|
| **The proposed method** | 0.76 | | | | 3.1 |
| | | 15.00 | 15.70 | 99.5 | 3.8 |
| | | 20.00 | 20.52 | 98.7 | 1.3 |
| | | 25.00 | 26.53 | 103.1 | 5.2 |
| **ELISA** | 0.74 | | | | 2.8 |
| | | 15.00 | 15.63 | 99.3 | 4.3 |
| | | 20.00 | 20.49 | 98.8 | 3.5 |
| | | 25.00 | 25.64 | 99.1 | 3.3 |
